# Right ventricular outflow tract morphology and its clinical significance for invasive procedures

**DOI:** 10.1038/s41598-025-06663-w

**Published:** 2025-07-09

**Authors:** Marcin Jakiel, Rafał Jakiel, Jakub Batko, Edyta Dyngosz, Maria Kurek, Karolina Gutkowska, Jakub Hołda, Filip Bolechała, Marcin Strona, Mateusz K. Hołda

**Affiliations:** 1 HEART – Heart Embryology and Anatomy Research Team, Department of Anatomy, Jagiellonian University Medical College, ul. Kopernika 12, Kraków, 31-034 Poland; 2 Department of Cardiovascular Diseases, Jagiellonian University Collegium Medicum, John Paul II Hospital in Kraków, Kraków, Poland; 3 Department of Endocrinology, Oncological Endocrinology, Nuclear Medicine and Internal Medicine, University Hospital in Kraków, Kraków, Poland; 4 Department of Cardiac Surgery and Transplantology, National Medical Institute of the Ministry of Interior and Administration, Warsaw, Poland; 5 Poland Health Care Center of the Ministry of Interior and Administration, Kraków, Poland; 6 Department of Forensic Medicine, Jagiellonian University Medical College, Kraków, Poland; 7 Division of Cardiovascular Sciences, The University of Manchester, Manchester, UK

**Keywords:** Right ventricular outflow track, Right ventricle, RVOT septal component, Subpulmonary infundibulum, Pulmonary root, Supraventricular crest, Septoparietal band, Ventricular arrhythmias, Anatomy, Cardiology

## Abstract

We aimed to evaluate in detail the right ventricular outflow tract (RVOT) morphology to enhance clinical procedures performed within this area. Two hundred twenty autopsied adult human hearts (37.2% females, 47.6 ± 18.0 years old) without macrostructural cardiac abnormalities were examined. The RVOT was macroscopically and morphometrically evaluated. The RVOT has a truncated cone shape with the elliptical RVOT base being significantly larger than its distal boundary (pulmonary root basal ring) (mean perimeter: 126.6 ± 27.7 vs. 73.2 ± 11.6 mm, *p* < 0.001). The right RVOT wall is the highest (30.5 ± 6.1 mm), followed by the anterior (29.4 ± 7.3 mm), left (27.3 ± 6.4 mm), and posterior (26.9 ± 6.5 mm). The RVOT may be divided into the free-standing thin-walled subpulmonary infundibulum and the septal component, which constitutes a significant portion of the RVOT posterior wall (37.3 ± 13.8%). The RVOT wall thickness demonstrated significant differences among the respective RVOT walls and across the RVOT levels. The length of the supraventricular crest is 29.1 ± 6.9 mm, and it narrows along its course (thickness at origin: 12.26 ± 2.9 mm vs. at termination: 9.2 ± 1.9 mm, *p* < 0.001). The length of the septoparietal band is 16.6 ± 3.9 mm, and its thickness remains relatively constant along its entire length (at origin: 6.9 ± 1.6 mm, at termination: 7.2 ± 2.4 mm, *p* = 0.124). Endocardial roughness of the RVOT, such as trabeculations, recesses, or muscular bridges, are present in all hearts within the subpulmonary infundibulum and in only 8.2% of the septal component. No significant differences between sexes were found in any of the measured RVOT parameters. In conclusion, this study provides a detailed definition, along with anatomical and structural analysis of the RVOT, highlighting its complex morphology and its significant role in cardiac functionality and clinical interventions.

**Supplementary Information:**

The online version contains supplementary material available at 10.1038/s41598-025-06663-w.

## Introduction

The right ventricular outflow tract (RVOT) was first described as being anatomically separated from the main portion of the right ventricle by Keith in 1924^1^. It develops from two primary embryological structures: the bulbus cordis and the conus arteriosus, which undergo significant remodeling and septation during embryogenesis to form the mature outflow tracts of the heart^2,3^. The RVOT may be macroscopically distinguished from the right ventricle inflow tract by the smaller size and different orientation of the muscle fibers (running in a parallel course from epicardium to endocardium), while maintaining a similar wall thickness^4^. The anatomical boundary between the right ventricle inflow and outflow tracts is shaped by the supraventricular crest^3^. At its distal end the RVOT is bounded by the basal ring of the pulmonary root^5,6^. The RVOT is a complex anatomical region of the heart that accommodates several important structures: the papillary muscles of the right ventricle, various types of tendinous cords, the trabeculae carneae of the right ventricle, septomarginal and septoparietal trabeculations, moderator band, and the subpulmonary infundibulum^7–10^. These structures collectively contribute to the RVOT’s function of directing blood flow from the right ventricle into the pulmonary circulation.

The RVOT is the most common site for the generation of ventricular arrhythmias arising in a heart without structural disease, including the most serious type, ventricular fibrillation^11–13^. The arrhythmias originating from the RVOT have diverse etiologies, including genetic predispositions, structural anomalies, or functional disturbances, and can affect patients across all age groups, ranging from young individuals without apparent heart disease to older adults with various cardiovascular conditions^14,15^. The treatment of RVOT arrhythmias involves a combination of pharmacological therapy and transcatheter procedural interventions^16^. Additionally, the RVOT may be used as an access point to ablate arrhythmias arising from adjacent cardiac structures, such as the left ventricular summit^17,18^. Finally, RVOT provides an access route for structural procedures within the pulmonary valve and pulmonary trunk^19^.

Despite its growing clinical importance, the RVOT area remains one of the least anatomically understood regions of the heart^3,20^. Meanwhile, gaining knowledge of the structure of this complex anatomical entity may enhance procedures such as electrocardiological anatomical mapping, catheter ablations, and device and lead implantations. Additionally, this understanding will help reduce complications, including RVOT perforation and damage to structures located within the RVOT^21–23^. Therefore, the aim of the current study is to evaluate in detail the RVOT architecture, with particular reference to the morphology of its endocardial surface and the structure of its walls. This will enable a better understanding of potential challenges and difficulties that may occur during cardiac procedures performed within this region, thereby increasing their effectiveness and safety.

## Materials and methods

This study was conducted at the Department of Anatomy of the Jagiellonian University Medical College in Krakow, Poland, and received approval from the Bioethical Committee of the Jagiellonian University, Krakow, Poland (No. 1072.6120.31.2019). The study protocol complied with the ethical guidelines of the 1975 Declaration of Helsinki.

We analyzed 220 hearts (37.2% female) obtained during forensic medical autopsies from adult donors (mean age: 47.6 ± 18.0 years, mean BMI: 26.4 ± 4.1 kg/m²) without macrostructural cardiac abnormalities. The exclusion criteria included heart trauma, history of heart surgeries or grafts, any visible severe macroscopic heart or vascular system pathologies found during the autopsy, and macroscopic signs of cadaver decomposition.

**Fig. 1:**
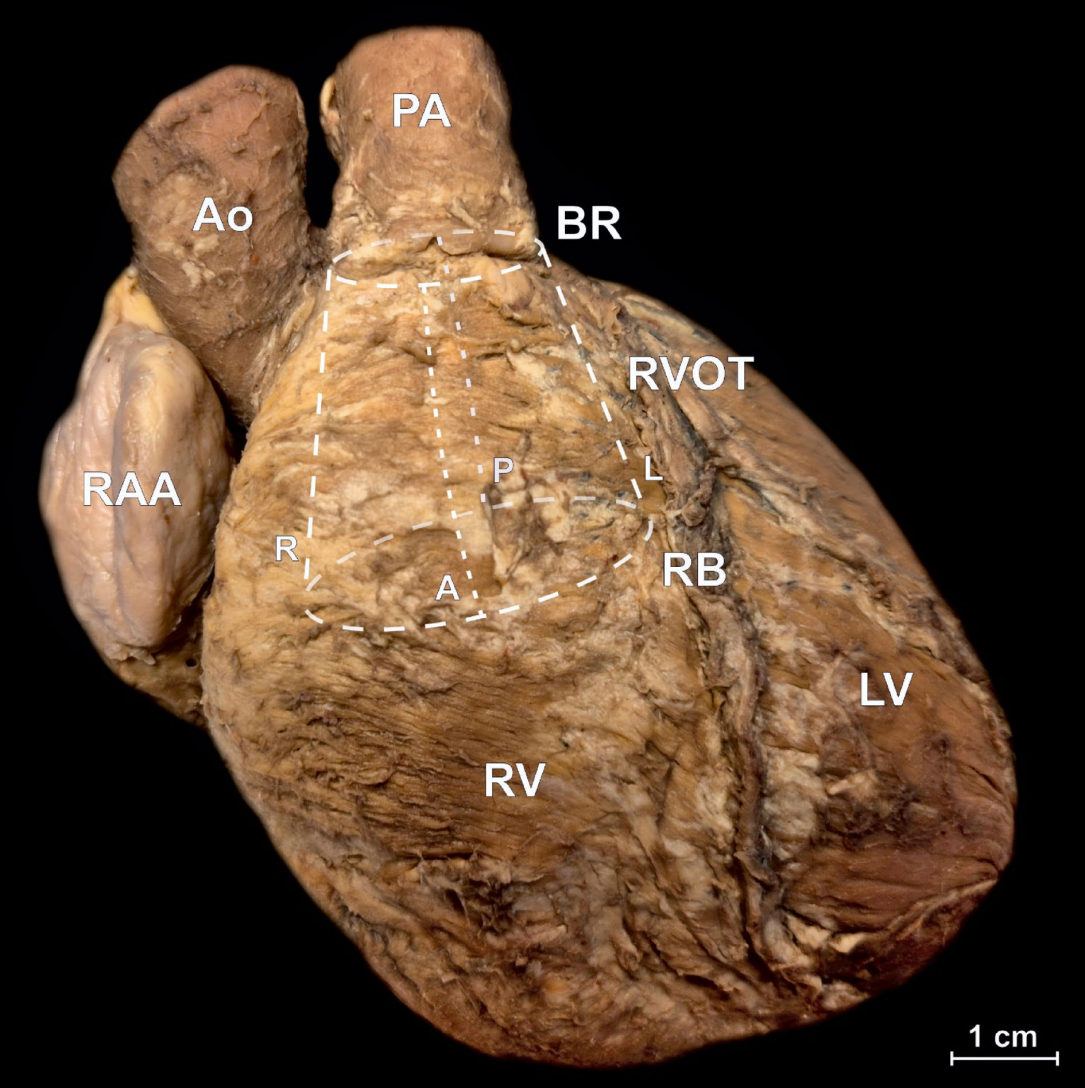
– Photograph of cadaveric heart specimen, anterior view of the heart in anatomical position. The location of right ventricular outflow tract (RVOT) is schematically marked. A – RVOT anterior periphery, Ao – aorta, BR – basal ring, L – RVOT left periphery, LV – left ventricle, P – RVOT posterior periphery, PA – pulmonary artery, R – RVOT right periphery, RB – RVOT base, RV – right ventricle, RAA – right atrial appendage.

**Fig. 2:**
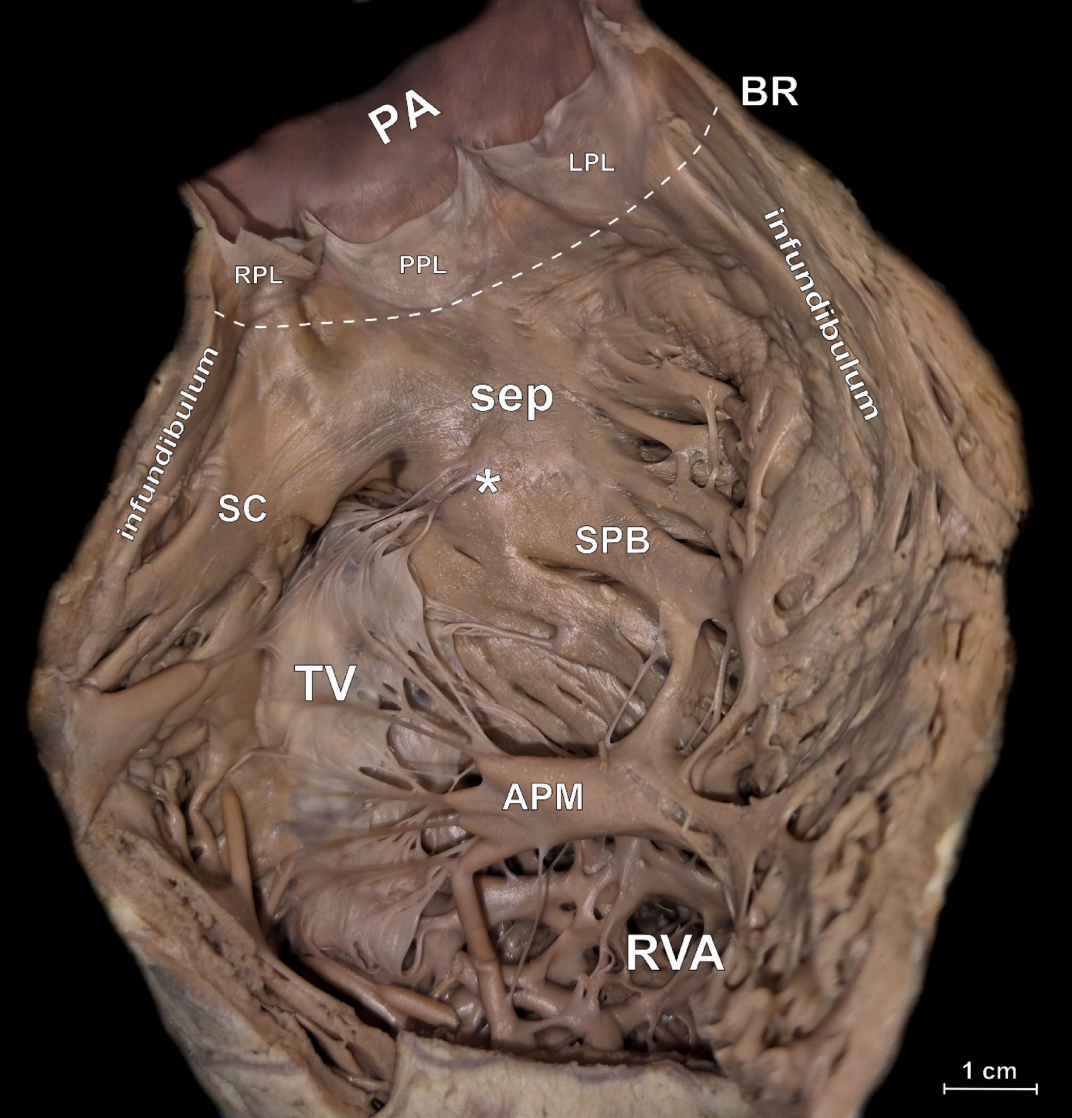
– Photograph of cadaveric heart specimen showing exposed posterior wall of right ventricular outflow tract (RVOT). White arrow indicates the area on left RVOT wall with different types of endocardial roughness. APM – anterior papillary muscle, BR – basal ring, LPL – left anterior pulmonary valve leaflet, PA – pulmonary artery, PPL – posterior pulmonary valve leaflet, RAVV – right atrioventricular valve, RPL – right anterior pulmonary valve leaflet, RVA – right ventricle apical part, SC – supraventricular crest, sep – RVOT septal component, SPB - septoparietal band, * – septal band/septal papillary muscle.

**Fig. 3:**
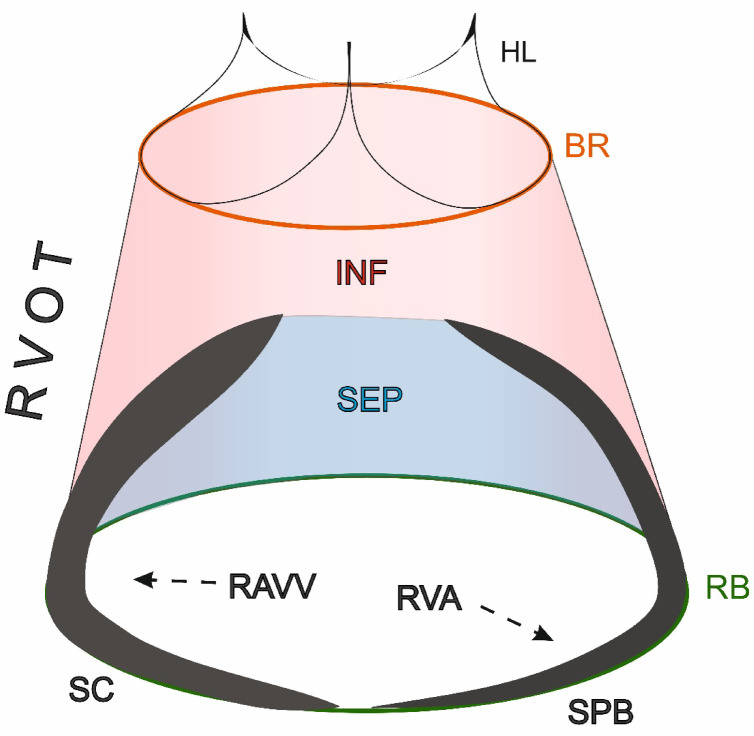
– Schematic picture of the right ventricular outflow tract (RVOT) with its main components. BR – basal ring, HL – hinge line of pulmonary valve leaflets, INF – subpulmonary infundibulum, RB – RVOT base, RAVV – right atrioventricular valve, RVA – right ventricle apical part, SC – supraventricular crest, SEP – RVOT septal component, SPB - septoparietal band.

Collected hearts were dissected from the chest cavity and fixed in a 10% formaldehyde solution. After 2 months of fixation, the RVOT was opened by cutting the right ventricle wall, pulmonary root, and pulmonary artery along their right peripheries. The following definitions were adopted to describe the studied area:

- RVOT – a part of the right ventricle, it is a tubular muscular structure in the shape of a truncated cone. It is bounded proximally (from the rest of the right ventricle) by a horizontal plane passing through the lowest point of the supraventricular crest (RVOT base) and distally (from the pulmonary root) by the basal ring of the pulmonary root (Fig. 1). The RVOT is divided into two parts: the septal component and the subpulmonary infundibulum (Figs. 2 and 3),

- RVOT septal component – the proximal part of the RVOT, it is a part of the interventricular septum that forms the postero-medial wall of the RVOT. It is delimited from the subpulmonary infundibulum by the initial sections of supraventricular crest (from the medial and posterior) and septoparietal band (from the anterior aspect) (Fig. 2),
- subpulmonary infundibulum – the funnel-shaped portion of the right ventricle that leads up to the pulmonary root. It is located above the trabeculated portion of the inflow and apical part of the right ventricle and the septal component of the RVOT and below the basal ring of the pulmonary root,
- supraventricular crest – a muscular ridge that arises from the bifurcation of its superior and inferior limbs located on the interventricular septum, travels along the superior part of the right atrioventricular annulus, and ends at the antero-lateral wall of the right ventricle,
- septoparietal band – the most prominent of the septoparietal trabeculations, it spans from the interventricular septum to the free wall of the right ventricle.

**Fig. 4:**
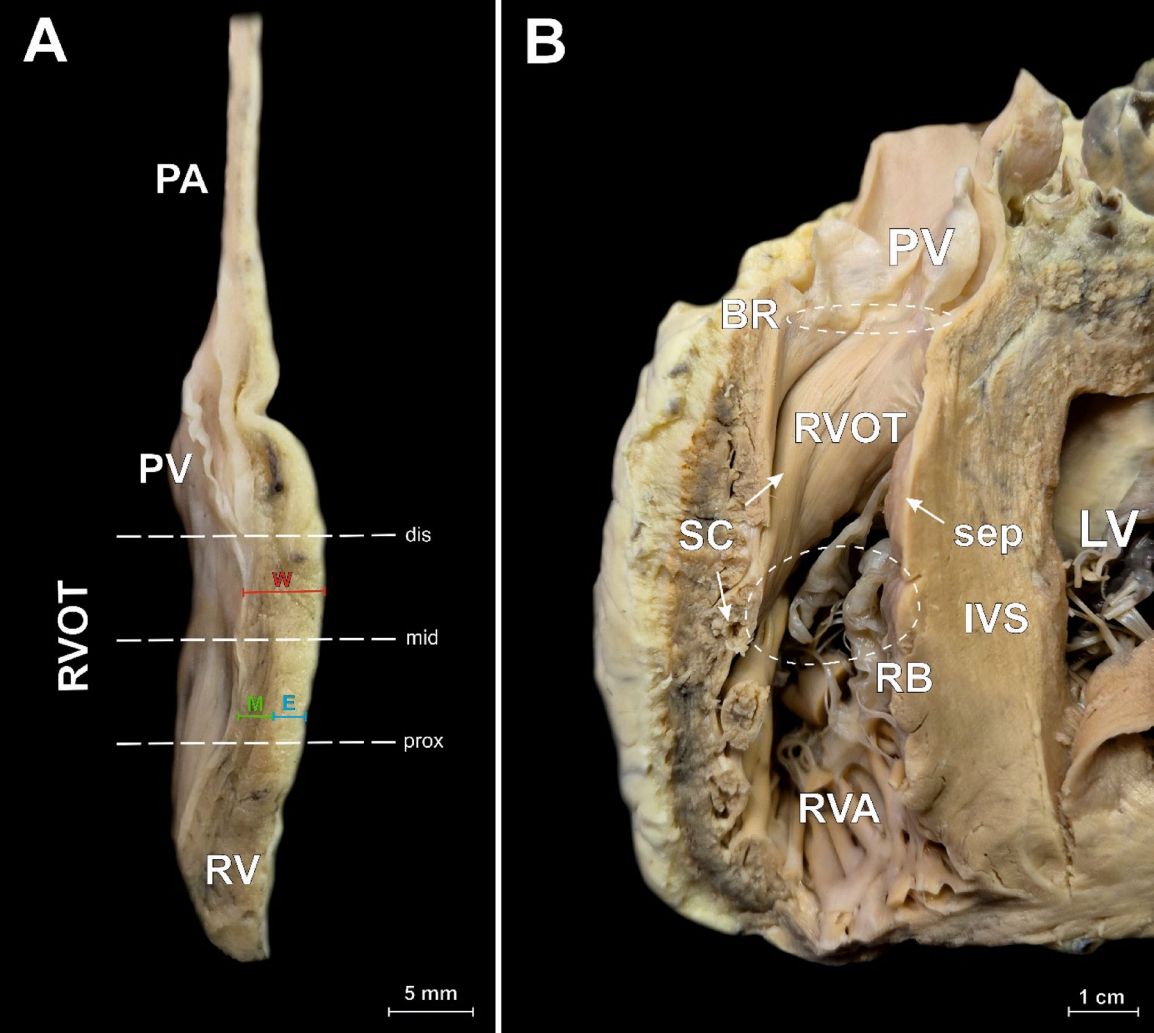
– Photographs of cadaveric heart specimens showing sections thorough right ventricular outflow tract (RVOT) walls. **A** – Magnified section thorough RVOT anterior wall showing measurements of wall thicknesses (W – whole wall thickness, M – myocardial thickness and E – epicardial adipose tissue thickness) at proximal (prox), middle (mid) and distal (dis) levels. **B** – Section thorough RVOT anterior and posterior walls showing differences in the walls composition. BR – basal ring, IVS – interventricular septum, LV – left ventricle, PA – pulmonary artery, PV – pulmonary valve, RB – RVOT base, RV – right ventricle, RVA – right ventricle apical part, SC – supraventricular crest, sep – RVOT septal component.

The following measurements were performed:

- RVOT base perimeter – measured as the sum of distances along right ventricle wall at the level of RVOT proximal boundary. The surface area of the RVOT base was calculated (assumed that this area is a simplified ellipse),
- basal ring perimeter (RVOT distal boundary) – a sum of distances measured between each leaflet nadir, obtained on a flat laid valve. The surface area of the basal ring was calculated (assumed that this area is a simplified ellipse),
- height of the RVOT – measured between RVOT base and basal ring of the pulmonary root along the middle of the RVOT anterior, posterior left and right walls,
- size of the RVOT septal component – maximum height of the septal component (from the RVOT base to the subpulmonary infundibulum boundary) and width of the RVOT septal component base were measured. The surface area of the RVOT septal component was calculated (for the calculations it was assumed that this area is a simplified half-ellipse),
- height of the subpulmonary infundibulum – measured along the middle of its anterior, posterior, left and right walls,
- supraventricular crest length and thickness (at its origin and termination). The types of the final ramifications of the supraventricular crest were also noted,
- septoparietal band length and thickness (at its origin and termination),
- whole-wall thickness (endocardium, myocardium and epicardial adipose tissue), myocardial thickness and epicardial adipose tissue thickness – measured at wall cross-sections along the subpulmonary infundibulum circumference (anterior, posterior, right and left walls) at its proximal, middle and distal levels (Fig. 4A). Please note that posterior wall of the RVOT corresponds mainly to the interventricular septum (RVOT septal component) where not all measurements were possible.

The macroscopic structure of the RVOT endocardial surface was also analyzed. The presence of the trabeculations, recesses and muscular bridges was evaluated and the depth of recesses was measured.

All observations and measurements were performed on the heart in the anatomically correct anatomical position. Linear dimensions were recorded with a 0.03-mm precision electronic caliper (YT-7201; YATO, Wroclaw, Poland) by two different investigators, and the mean of two values was calculated and reported as the final value. Statistical analyses were performed using STATISTICA 13.1 software for Windows (StatSoft Inc., Tulsa, OK, USA). Categorical variables were presented as numbers (n) or percentages, and quantitative variables as means with standard deviations. Normal distribution was assessed using the Shapiro-Wilk test. Differences between normally distributed quantitative parameters were evaluated with Student’s t-test, while non-normally distributed quantitative data were analyzed using the Mann-Whitney U test. Differences between categorical variables were determined using the chi-square test of independence. Differences between more than two groups were also assessed. For normally distributed data one-way analysis of variance (one-way ANOVA) was used with Tukey’s post hoc test, if ANOVA results were statistically significant. For non-normally distributed data Kruskal and Wallis’ test was used with Dunn’s post hoc test if the Kruskal and Wallis’s test results were statistically significant. Correlation coefficients were calculated to assess statistical dependence between measured parameters. A p-value of < 0.05 was considered statistically significant.

## Results

### Geometry of the RVOT

Table 1 presents the results of geometric measurements performed for all RVOT components. The RVOT has a truncated cone shape with the elliptical RVOT base being significantly larger than the basal ring (mean perimeter: 126.6 ± 27.7 vs. 73.2 ± 11.6 mm, *p* < 0.001) (Figs. 1 and 3). The RVOT walls are not of equal height, with the right wall being the highest (30.5 ± 6.1 mm), followed by the anterior (29.4 ± 7.3 mm), left (27.3 ± 6.4 mm), and posterior (26.9 ± 6.5 mm) (p-value ANOVA < 0.001). The size of the subpulmonary infundibulum, which represents the free-standing thin wall of the RVOT, is significantly smaller than the RVOT in its posterior aspect, where it shares the RVOT wall with the septal component (15.3 ± 6.5 vs. 26.9 ± 6.5 mm, *p* < 0.001, Table 1). Both the left and right walls of the infundibulum are also lower than those of the RVOT due to the presence of the supraventricular crest and septoparietal band located at the base of the RVOT (Table 1, *p* < 0.001), while the anterior wall of the RVOT is fully occupied by the subpulmonary infundibulum. The septal component is of considerable size (Table 1; Fig. 2) and constitutes a significant portion of the RVOT posterior wall (37.3 ± 13.8%).

**Table 1:** – Results of measurements performed for right ventricular outflow tract (RVOT) components (mean ± SD [min-max range]) and broken down by sex.

Parameter		Total (*n* = 220)	Males (*n* = 138)	Females (*n* = 82)	*p*-value males vs. females
RVOT base perimeter (mm)		126.6 ± 27.7 [73.0-229.4]	127.0 ± 30.6 [73.0-229.4]	123.9 ± 22.1 [84.3-188.9]	0.584
basal ring perimeter (mm)		73.2 ± 11.6 [27.1–95.9]	73.1 ± 10.5 [27.1–92.3]	73.8 ± 14.2 [47.8–95.9]	0.870
RVOT base surface area (mm^2^)		1336.8 ± 600.5 [424.3-4190.1]	1349.0 ± 633.1 [424.3-4190.1]	1259.4 ± 472.1 [565.4–2842.0]	0.462
basal ring surface area (mm^2^)		437.6 ± 128.4 [58.6-732.2]	437.2 ± 120.6 [58.6-677.6]	442.4 ± 138.8 [182.6-732.2]	0.951
height of the RVOT wall (mm)	anterior	29.4 ± 7.3 [13.1–50.1]	29.0 ± 7.5 [13.1–50.1]	30.5 ± 7.2 [20.2–50.2]	0.155
	posterior	26.9 ± 6.5 [10.5–47.7]	26.7 ± 6.7 [10.5–47.7]	27.2 ± 5.6 [15.3–38.4]	0.160
	left	27.3 ± 6.4 [13.6–47.6]	27.1 ± 6.6 [13.6–47.6]	27.6 ± 6.1 [15.3–42.1]	0.336
	right	30.5 ± 6.1 [17.4–52.7]	30.4 ± 6.2 [17.4–52.7]	31.1 ± 6.0 [17.7–50.7]	0.246
height of the subpulmonary infundibulum (mm)	anterior	29.4 ± 7.3 [13.1–50.1]	29.0 ± 7.5 [13.1–50.1]	30.5 ± 7.2 [20.2–50.2]	0.155
	posterior	15.3 ± 6.5 [2.9–34.5]	15.1 ± 6.6 [2.9–34.2]	14.9 ± 5.3 [6.4–25.3]	0.435
	left	20.4 ± 6.6 [6.6–37.2]	20.4 ± 7.0 [6.6–37.2]	19.4 ± 6.0 [11.7–36.1]	0.700
	right	19.7 ± 6.3 [6.7–40.4]	19.6 ± 6.2 [6.7–36.7]	21.7 ± 6.6 [12.6–40.4]	0.079
RVOT septal component height (mm)		12.7 ± 4.8 [4.4–31.8]	12.7 ± 4.7 [4.4–31.8]	12.7 ± 5.3 [7.0-30.4]	0.474
RVOT septal component width (mm)		40.3 ± 8.8 [20.5–73.1]	40.5 ± 9.1 [20.5–73.1]	39.3 ± 7.1 [26.9–60.2]	0.211
RVOT septal component surface area (mm^2^)		393.7 ± 178.6 [102.4-1094.9]	397.6 ± 180.5 [102.4-1094.9]	374.6 ± 170.8 [163.5-843.2]	0.270
supraventricular crest length (mm)		29.1 ± 6.9 [11.4–51.2]	29.1 ± 7.0 [11.4–51.2]	29.0 ± 6.8 [14.3–43.7]	0.457
supraventricular crest thickness at origin (mm)		12.3 ± 2.9 [5.4–22.2]	12.3 ± 3.0 [6.5–22.2]	11.1 ± 2.6 [5.4–20.6]	0.062
supraventricular crest thickness at termination (mm)		9.2 ± 1.9 [4.7–15.8]	9.3 ± 1.9 [5.2–15.8]	9.0 ± 2.0 [4.7–13.7]	0.160
septoparietal band length (mm)		16.6 ± 3.9 [4.5–36.7]	16.2 ± 3.7 [4.5–26.6]	17.5 ± 4.9 [12.2–36.7]	0.054
septoparietal band thickness at origin (mm)		6.9 ± 1.6 [3.1–11.7]	7.0 ± 1.6 [3.1–11.7]	6.7 ± 1.7 [3.1–10.7]	0.240
septoparietal band thickness at termination (mm)		7.2 ± 2.4 [2.6–14.8]	7.2 ± 2.3 [2.6–14.8]	7.5 ± 2.8 [3.7–13.8]	0.368

No significant differences between sexes were found in any of the measured RVOT parameters (Table 1, all *p* > 0.05). Table 2 presents the Pearson correlation matrix illustrating relationships both among the measured RVOT parameters themselves and between these parameters and anthropometric characteristics. As demonstrated in Table 2, no significant correlations were found between RVOT parameters and age, BMI, or BSA. A statistically significant but weak positive correlation was observed only between heart weight and the height of the anterior RVOT wall (or subpulmonary infundibulum) (*r* = 0.202, *p* = 0.007). On the other hand, numerous significant positive correlations were observed among the various RVOT parameters. In general, greater RVOT and subpulmonary infundibulum heights at one measurement site were associated with greater heights at other sites, as well as with a larger septal component area (see Table 2).

**Table 2:** Correlaton matrix of pearson correlation coefficient (r values reported, *n* = 220). Bold indicates significant corrrelations (p-value < 0.05).

Parameter		RVOT base perimeter	basal ring perimeter	RVOT base surface area	basal ring surface area	height of the RVOT wall				height of the subpulmonary infundibulum				RVOT septal component height
						anterior	posterior	left	right	anterior	posterior	left	right	
RVOT base perimeter		1.000												
basal ring perimeter		**0.523**	1.000											
RVOT base surface area		1.000	**0.554**	1.000										
basal ring surface area		**0.567**	1.000	**0.455**	1.000									
height of the RVOT wall	anterior	**0.216**	0.060	**0.216**	0.060	1.000								
	posterior	0.147	0.009	0.147	0.009	**0.860**	1.000							
	left	0.173	-0.005	0.173	-0.005	**0.873**	**0.950**	1.000						
	right	**0.273**	0.142	**0.273**	0.142	**0.648**	**0.700**	**0.734**	1.000					
height of the subpulmonary infundibulum	anterior	**0.216**	0.060	**0.216**	0.060	**1.000**	**0.860**	**0.873**	**0.648**	1.000				
	posterior	0.171	0.110	0.171	0.112	**0.654**	**0.745**	**0.747**	**0.457**	**0.654**	1.000			
	left	0.144	-0.118	0.144	-0.118	**0.759**	**0.858**	**0.866**	**0.638**	**0.759**	**0.634**	1.000		
	right	0.135	-0.024	0.135	-0.024	**0.575**	**0.717**	**0.749**	**0.937**	**0.575**	**0.326**	**0.661**	1.000	
RVOT septal component height		0.069	0.064	0.069	0.064	0.174	0.199	**0.207**	**0.327**	0.174	**-0.357**	0.148	**0.375**	1.000
RVOT septal component widht		1.000	**0.517**	**0.986**	**0.519**	**0.216**	0.147	**0.207**	**0.273**	**0.216**	0.169	0.144	0.135	0.069
RVOT septal component surface area		**0.616**	**0.259**	**0.567**	**0.534**	**0.558**	**0.648**	0.173	**0.475**	**0.558**	**0.544**	**0.615**	**0.492**	0.144
supraventricular crest length		**0.460**	**0.318**	**0.460**	**0.318**	**0.329**	**0.228**	**0.254**	**0.527**	**0.329**	0.054	**0.207**	**0.456**	**0.273**
supraventricular crest thickness at origin		-0.055	0.272	-0.055	0.272	0.160	0.122	0.165	0.062	0.160	0.259	0.035	0.122	-0.050
supraventricular crest thickness at termination		0.059	0.184	0.059	0.184	0.066	0.036	0.077	0.143	0.066	0.087	0.102	-0.004	0.010
septoparietal band length		-0.057	0.041	-0.057	0.040	-0.032	-0.104	-0.057	0.020	-0.032	-0.168	-0.064	0.064	0.066
septoparietal band thickness at origin		0.010	-0.036	0.010	-0.036	-0.060	**-0.273**	-0.145	-0.123	-0.06	**-0.266**	**-0.256**	-0.162	0.078
septoparietal band thickness at termination		-0.079	0.129	0.079	0.129	-0.026	-0.123	-0.064	-0.048	-0.026	-0.042	**-0.399**	0.160	-0.051
age		0.073	-0.091	0.073	-0.091	0.032	0.129	0.112	0.141	0.032	0.148	0.146	0.070	-0.109
BMI		0.094	0.033	0.094	0.033	-0.017	-0.121	-0.104	-0.100	-0.017	0.014	-0.094	-0.041	-0.137
BSA		0.161	0.088	0.161	0.088	-0.021	-0.097	-0.009	-0.130	-0.021	-0.065	-0.038	-0.098	-0.055
heart weight		0.132	-0.005	0.132	-0.005	**0.202**	0.153	0.143	0.107	**0.202**	0.167	0.192	-0.098	-0.012
heart perimeter		-0.070	-0.145	-0.070	-0.145	-0.047	0.093	0.033	-0.033	-0.047	-0.144	0.204	-0.030	0.021

Parameter		RVOT septal component width	RVOT septal component surface area	supra-ventricular crest length	supra-ventricular crest thickness at origin	supra-ventricular crest thickness at termination	septo-parietal band length	septo-parietal band thickness at origin	septo-parietal band thickness at termination	age	BMI	BSA	heart weight	heart perimeter
RVOT base perimeter														
basal ring perimeter														
RVOT base surface area														
basal ring surface area														
height of the RVOT wall	anterior													
	posterior													
	left													
	right													
height of the subpulmonary infundibulum	anterior													
	posterior													
	left													
	right													
RVOT septal component height														
RVOT septal component widht		1.000												
RVOT septal component surface area		**0.616**	1.000											
supraventricular crest length		**0.459**	0.108	1.000										
supraventricular crest thickness at origin		-0.057	0.114	-0.128	1.000									
supraventricular crest thickness at termination		0.059	0.081	0.031	0.005	1.000								
septoparietal band length		0.057	-0.172	0.197	0.012	-0.172	1.000							
septoparietal band thickness at origin		0.010	0.017	0.064	0.015	0.017	**0.332**	1.000						
septoparietal band thickness at termination		-0.079	0.094	-0.068	0.138	0.094	0.084	**0.436**	1.000					
age		0.073	0.071	0.039	-0.189	0.075	-0.150	-0.025	0.021	1.000				
BMI		0.094	-0.037	-0.046	-0.005	0.058	0.064	0.082	0.063	0.125	1.000			
BSA		0.161	-0.015	-0.029	0.120	0.034	0.015	0.054	-0.059	-0.124	**0.614**	1.000		
heart weight		0.132	0.146	0.023	0.119	0.034	-0.003	0.050	-0.034	**0.301**	**0.368**	**0.484**	1.000	
heart perimeter		-0.065	-0.007	-0.001	0.055	0.087	-0.092	-0.115	0.005	**0.341**	**0.261**	**0.322**	**0.510**	1.000

### Thickness of the RVOT walls

The measured whole-wall thickness of the RVOT walls demonstrated significant differences both among the respective RVOT walls and across the RVOT levels (proximal vs. middle vs. distal) (Table 3; Fig. 4). Generally, the posterior wall is the thickest, followed by the right wall, while the anterior and left walls exhibit similar thickness (Table 3 and Supplementary Table 1). The posterior RVOT wall is entirely composed of myocardium, with none or a negligible amount of epicardial adipose tissue, and its thickness tapers from the RVOT base toward the distal direction (Table 3 and Supplementary Tables 1, 10.3 ± 3.8 vs. 8.7 ± 3.5 mm, *p* = 0.003, Fig. 4B). For the remaining RVOT walls, the myocardial layer is relatively uniform both across the walls and levels, averaging 4–5 mm in thickness with significant differences proximally, while in the middle section only between the right and left periphery (Table 3 and Supplementary Table 1). The right RVOT wall contains the most epicardial adipose tissue, followed by the left and anterior walls (p-value ANOVA < 0.001), with the adipose tissue being more prominently distributed at the distal levels compared to the proximal levels (p-value ANOVA < 0.001, Table 3 and Supplementary Table 1). No significant differences in measured tissue thicknesses were observed between sexes and no significant correlations were observed with anthropometric parameters (all *p* < 0.05).

**Table 3:** Whole-wall, myocardial and epicardial adipose tissue thickness of the right ventricular outflow tract (RVOT) walls (mean ± SD [min-max range]).

Parameter	Level	RVOT wall			
		anterior	posterior	left	right
Whole-wall thickness (mm)	proximal	5.7 ± 2.3 [1.4–23.0]	-*	5.6 ± 2.4 [2.3–18.3]	7.9 ± 2.6 [2.3–15.5]
	middle	6.2 ± 2.0 [1.6–15.5]	10.3 ± 3.8 [1.6–20.0]	6.0 ± 2.2 [2.4–14.8]	7.4 ± 2.7 [1.9–17.2]
	distal	5.9 ± 1.8 [2.0-12.5]	8.7 ± 3.5 [1.6–17.5]	6.1 ± 2.2 [2.3–18.2]	8.6 ± 2.7 [3.5–17.3]
Myocardial thickness (mm)	proximal	4.7 ± 1.4 [1.4–12.1]	-*	4.0 ± 1.4 [1.4–9.8]	5.4 ± 1.6 [1.9–10.4]
	middle	4.7 ± 1.3 [1.6–14.3]	10.3 ± 3.8 [1.6–20.0]	4.3 ± 1.2 [1.5–7.4]	5.3 ± 1.5 [2.0-10.7]
	distal	4.2 ± 1.2 [1.8–8.8]	8.7 ± 3.5 [1.6–17.5]	4.3 ± 1.2 [1.8–8.7]	4.2 ± 1.4 [1.7–8.5]
Epicardial adipose tissue thickness (mm)	proximal	1.0 ± 1.6 [0.0-10.9]	none	1.7 ± 1.7 [0.0–11.0]	2.6 ± 2.0 [0.0-11.3]
	middle	1.6 ± 1.5 [0.0-7.9]	none	1.8 ± 1.7 [0.0–12.0]	3.2 ± 2.3 [0.0-11.9]
	distal	1.7 ± 1.3 [0.0-6.3]	none	1.9 ± 1.8 [0.0-14.3]	3.4 ± 2.1 [0.0-12.8]

* - posterior wall of the RVOT corresponds to the interventricular septum and RVOT septal component, where not all measurements were possible at all levels and where epicardial adipose tissue is absent.

Statistical comparisons between different measurement points (ANOVA) are presented in Supplementary Table 1.

### Supraventricular crest

The supraventricular crest was present in all cases. It arises from the interventricular septum (posterior RVOT wall), runs to the right, and terminates in the anterior RVOT wall (Figs. 2 and 4B). The total length of the supraventricular crest is 29.1 ± 6.9 mm, and it narrows along its course (thickness at origin: 12.26 ± 2.9 mm vs. at termination: 9.2 ± 1.9 mm, *p* < 0.001, Table 1). While the origin of the supraventricular crest is consistent, its termination exhibits various patterns. In 61.0% of hearts, the terminal part of the supraventricular crest did not divide, ending as a compact, single muscular band. In 17.3% of cases, a dichotomous division of the supraventricular crest was observed. In the remaining 21.7% of cases, it divided into several branches, with recesses, tissue bridges, and strands noted between the branches. No significant differences in supraventricular crest morphology were observed between sexes, and no significant correlations with anthropometric parameters were identified. Additionally, significant positive correlations were found between supraventricular crest length and almost all other measured RVOT parameters, whereas no such correlations were observed for supraventricular crest thickness (see Table 2).

### Septoparietal band

Septoparietal band is the most prominent of the septoparietal trabeculations and may be found in all studied hearts. It arises from the posterior wall of the RVOT wall (Fig. 2), runs anteriorly, and terminates in the anterior RVOT wall where it meets the supraventricular crest terminal branches. The total length of the septoparietal band is 16.6 ± 3.9 mm, and its thickness is relatively constant along its entire length (thickness at origin: 6.9 ± 1.6 mm vs. at termination: 7.2 ± 2.4 mm, *p* = 0.124, Table 1). Neither significant differences in septoparietal band size between sexes (Table 1), nor significant correlations with anthropometric parameters were observed (Table 2).

### Trabeculations, recesses and muscular bridges

**Fig. 5:**
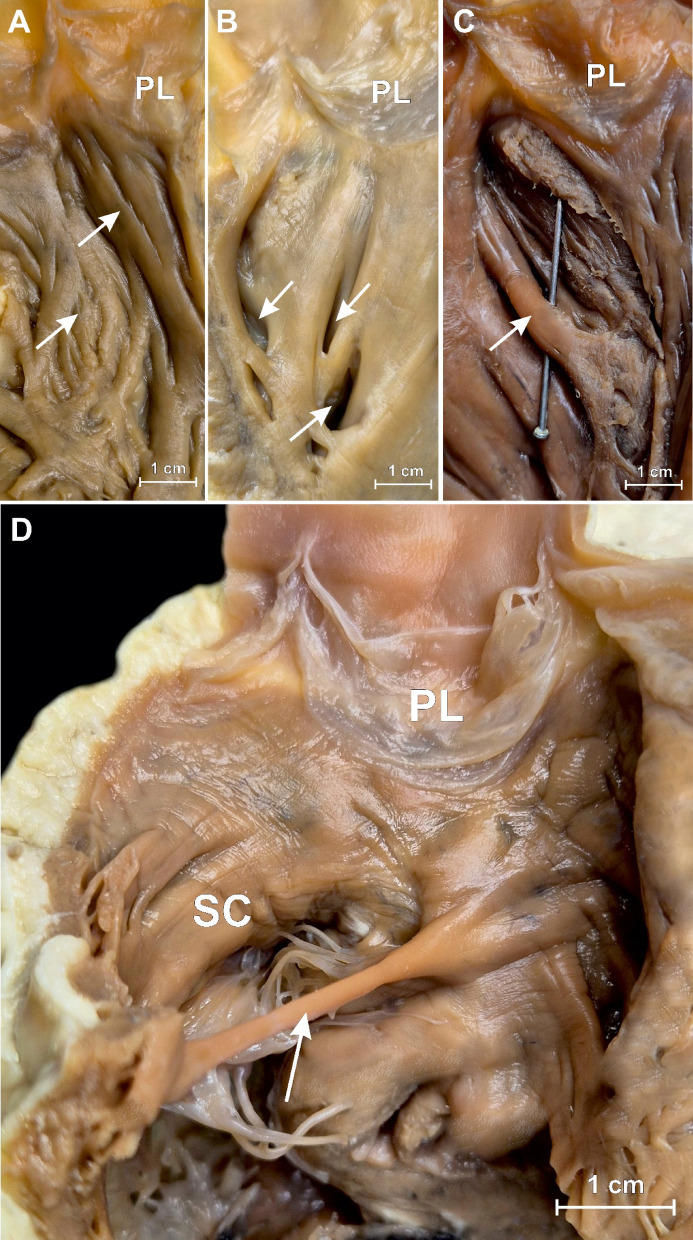
– Photographs of cadaveric heart specimens showing endocardial roughness of the right ventricular outflow tract (RVOT) walls. **A** – trabeculations. **B** – recesses. **C** – muscular bridges, **D** – tissue bridge connecting opposite RVOT walls.

Endocardial roughness of the RVOT, such as trabeculations, recesses, or muscular bridges (Fig. 5), may be observed in all hearts within the subpulmonary infundibulum, but are no evenly distributed. The anterior wall of the RVOT is smooth in 5.9% of cases, while the left and right walls are smooth in 29.1% and 33.6% of cases, respectively. Trabeculations (Fig. 5A) are the most commonly observed feature of infundibular roughness, present in 96.0% of all hearts. They are predominantly located on the anterior wall (94.1%), followed by the left (35.5%) and right (21.8%) walls of the RVOT. Recesses (Fig. 5B) are observed exclusively on the left and right RVOT walls, occurring in 62.3% and 59.5% of cases, respectively, with a mean depth of 11.1 ± 5.2 mm. Muscular bridges (Fig. 5C) are rare, present in only 3.8% of cases, and are restricted to the left RVOT wall. In 1.4% of cases, a tissue bridge connecting opposite RVOT walls can be detected (Fig. 5D). The septal component is typically free of trabeculations; however, muscular bridges or recesses are occasionally observed, each occurring in 4.1% of hearts. The posterior wall of the subpulmonary infundibulum is generally smooth. No significant differences in endocardial roughness distribution were observed between sexes.

## Discussion

This study presents a comprehensive anatomical and morphometric evaluation of the RVOT based on 220 autopsied human hearts. We provided a refined anatomical definition of the RVOT and characterized its structural components, including the septal component and subpulmonary infundibulum. Our results demonstrate significant differences in RVOT wall height and thickness, with the anterior wall being the thinnest and the posterior wall the thickest. Additionally, we observed variable distribution of endocardial features such as trabeculations, recesses, and muscular bridges, predominantly within the subpulmonary infundibulum. Importantly, no significant differences were found in RVOT parameters between sexes, and anthropometric variables such as age, body mass index, and body surface area did not correlate with RVOT measurements. A significant positive correlation was noted only between heart weight and the height of the anterior RVOT wall. These findings highlight the anatomical variability of the RVOT, which may have direct clinical relevance for planning and performing electrophysiological and interventional procedures. Overall, we believe that our study enhances the foundational anatomical knowledge necessary to improve safety and precision in RVOT-targeted therapies.

Previous studies have proposed various approaches to defining the RVOT. Unfortunately, even the latest research reveals a lack of precision in these definitions. For example, Haddad et al. defined the RVOT as the region extending from the septomarginal band to the pulmonary valve^24^. Israel et al. described the moderator band as a structure separating the inflow and apical parts of the right ventricle^25^. They further categorize the right ventricle into the inflow portion and the RVOT, distinguishing within the RVOT a subpulmonary infundibulum (also termed the high RVOT)^25^. Kaczynska et al. provided a simpler definition, locating the RVOT between the pulmonary valve and the level of the upper part of the supraventricular crest^26^. Some clinical studies, on the other hand, define the RVOT proximally by the superior aspect of the tricuspid annulus and distally by the pulmonary valve^13^. In our opinion, using the septomarginal (or moderator) band as a reference point for the proximal boundary of the RVOT may introduce potential errors, particularly by overlooking the supraventricular crest, which separates the inflow and outflow pathways of the right ventricle. Similarly, relating the proximal RVOT boundary constraint to the tricuspid ring, while close to reality, is inaccurate because the plane of the lower RVOT boundary lies in a different plane from the tricuspid ring itself. Furthermore, the term “pulmonary valve” is imprecise. Therefore, in the current study we propose a more precise definition of the RVOT. Specifically, the RVOT is a tubular muscular structure shaped like a truncated cone, bounded proximally (relative to the rest of the right ventricle) by a horizontal plane passing through the lowest point of the supraventricular crest (the RVOT base) and distally (relative to the pulmonary root) by the basal ring of the pulmonary root. This refined definition aims to resolve ambiguities in prior descriptions and provide a more anatomically accurate framework for clinical and research applications.

The RVOT consists of two primary components: the septal component, which forms the postero-medial wall, and the subpulmonary infundibulum, a funnel-shaped structure leading to the pulmonary valve. These anatomical subdivisions, with their distinct structural features—such as wall composition, proximity to adjacent cardiac structures, and variations in endocardial surface roughness—highlight the RVOT’s functional and clinical significance. A detailed delineation of its boundaries and internal architecture provides valuable insights into its role in both physiological blood flow and pathological conditions. For instance, ventricular arrhythmia foci can involve either the infundibulum or the septal component of the RVOT, enabling targeted arrhythmia ablation within either wall^27^. Further studies providing a detailed description of the anatomy and surrounding structures of the RVOT septal component are highly anticipated.

The thorough anatomical characterization of the RVOT components offers valuable insights for clinicians performing interventional procedures. The identification of specific geometric parameters, such as RVOT walls height, thickness, and the shape of the RVOT base and basal ring, enhances our understanding of this region’s variability. These findings are crucial for optimizing catheter positioning during ablation therapy and lead placement for pacemakers and defibrillators. The supraventricular crest is one of the crucial structures within the RVOT. However, descriptions of this structure in the literature are often inconsistent. Some authors use the terms ‘supraventricular crest’ and ‘ventriculo-infundibular fold’ interchangeably^25,28^. Although numerous studies link the supraventricular crest with other structures in the right ventricle (e.g., the moderator band, septal papillary muscle, and parietal band), as noted by other authors and confirmed by our findings, there is no consistent continuity between these structures^3,29,30^. Wafae et al. report that the supraventricular crest is present in all hearts^29^consistent with our observations. Another landmark structure investigated in the current study is the septoparietal band, a feature whose definition and descriptions in the literature are often ambiguous and confusing. The septoparietal band is a completely separate structure from other right ventricular entities. It is the most prominent of the septoparietal trabeculations, forming the left and anterior base of the RVOT and separating the outflow tract from the apical part of the right ventricle. Both the supraventricular crest and the septoparietal band play significant roles in right ventricular contraction^9^.

The presence of trabeculations or other forms of endocardial roughness within the RVOT has been a topic of debate among many authors. While some studies deny their existence^3,20^, others vaguely describe them as usually absent^24^. Individual descriptions suggest that trabeculations on the anterior RVOT wall extend to the uppermost region without detailing their distribution^25^. In contrast, our study found that anterior wall trabeculations were completely absent in only 5.9% of cases, with various distributions of endocardial roughness such as recesses and myocardial bridges in the remainder (Fig. 5). These findings indicate that defining the RVOT or differentiating it from the apical region of the right ventricle based solely on trabeculations is unreliable^10,28^. The identification of regions prone to endocardial roughness, such as trabeculations and recesses within the subpulmonary infundibulum, may serve as potential substrates for arrhythmogenesis^31^. Understanding the distribution and prevalence of these features is crucial for arrhythmia mapping and ablation strategies. For instance, the high prevalence of trabeculations on the anterior wall and the presence of deep recesses on the left and right RVOT walls may require meticulous exploration during electrophysiological procedures. Furthermore, such endocardial roughness can complicate ablation by obstructing catheters and hindering their contact with the arrhythmogenic tissue. Finally, the tissue bridge connecting opposite sides of the RVOT, revealed in this study and present in 1.4% of cases (Fig. 5D), may pose a significant risk during procedures performed within the RVOT and potentially hinder access to the pulmonary root.

Our study’s findings on RVOT walls thickness (Table 3) are consistent with previous literature^10,32^. The anterior wall of the RVOT, being the thinnest, presents the greatest risk of perforation and complications, such as cardiac tamponade, during invasive procedures^25^. This is particularly relevant in the context of Brugada syndrome, where catheter ablation is increasingly used as a therapeutic option. Although ablation in Brugada syndrome is usually performed via the epicardial route, it typically involves the anterior wall of the RVOT^15,33^which is also the thinnest and structurally most vulnerable part of the outflow tract. This anatomical characteristic raises specific safety concerns. In cases where combined epicardial and endocardial mapping or ablation is required, the risk of thermal injury or perforation increases significantly. Furthermore, the thinness of the anterior wall necessitates meticulous planning and adjustment of procedural parameters, such as energy delivery, contact force, and lesion duration, to prevent complications. The significant thickness of the posterior wall, composed predominantly of myocardium, underscores its resilience and suitability as a target for certain procedures, whereas the thinner anterior and lateral walls require careful handling. Reports on RVOT arrhythmia ablation via the anterior cardiac veins highlight the thinner fat layers on the left and anterior sides of the RVOT as favorable for access^34^. Moreover, the epicardial adipose tissue covering the RVOT may play a critical role not only in coronary artery disease but also as a contributor to arrhythmias^35–38^. Therefore, its uneven distribution around the RVOT walls may represent an important pathophysiological factor in these clinical entities.

In idiopathic ventricular arrhythmias, the site of origin within the RVOT can vary considerably. The mid-posterior septal region is among the most common locations^39^. However, electrocardiography does not always clearly indicate the precise location requiring ablation. Therefore, accurate identification of the arrhythmia site during the procedure is crucial^40^. A thorough understanding of the anatomical variability of the RVOT enables a more personalized approach, which may improve the effectiveness of ablation. In some cases, arrhythmogenic foci are located within or near the pulmonary valve leaflets. In such situations, the RVOT serves as the access route for catheters and other equipment used during the procedure^39^. These anatomical considerations are also important in the treatment of life-threatening arrhythmias in patients with arrhythmogenic right ventricular dysplasia, particularly in the early stages of the disease. At that time, structural abnormalities may not yet be evident, but arrhythmias may already arise from various regions within the RVOT. Recognizing this variability is essential for effective and safe ablation therapy^41^.

The RVOT is a highly complex three-dimensional structure that cannot be adequately visualized, defined, or measured in a single plane clinically. High-resolution imaging modalities, such as echocardiography, cardiac magnetic resonance imaging, and computed tomography, are essential for visualizing RVOT anatomy^28^. The detailed morphometric characteristics of the RVOT provided in the current study can contribute to the refinement of imaging protocols, enabling more precise delineation of the RVOT’s boundaries and internal structures. The variability in RVOT wall thickness and the presence of trabeculations and recesses highlight potential challenges in accurately identifying critical landmarks during clinical imaging. For example, the presence of trabeculations predominantly on the anterior wall and recesses on the left and right walls may obscure precise measurements or landmarks. Advanced imaging techniques, such as 3D echocardiography, 4D flow magnetic resonance or magnetic resonance imaging with tissue tagging, may improve the visualization of these structures^42,43^.

Several limitation of this study may be indicated. As this is a cadaveric study we were not able to assess the physiological changes in the RVOT components morphometric parameters during the cardiac cycle. Moreover, the tissue fixation in the paraformaldehyde solution may influence the morphometric observations. Nevertheless, our previous studies have verified that using formaldehyde for hearts preservation did not cause important changes in cardiac dimensions^44,45^. Furthermore, only hearts without significant structural diseases were included into analysis, thus the current study may serve only as a knowledge base about the unaffected structure of the RVOT. Finally, our study evaluated only those parameters that could be accurately determined on autopsied material, excluding some important elements of full spatial geometry of the RVOT. Despite above mentioned limitations, we believe that our study contributes to the growing body of knowledge on RVOT anatomy, offering practical insights that can improve the precision, safety, and outcomes of various cardiac procedures, thereby addressing a critical gap in the anatomical understanding of this vital cardiac region. Additional imaging studies (both clinical and experimental) are required to analyze the RVOT geometry in a broader perspective. Prospective studies combining high-resolution imaging with clinical outcomes data can provide deeper insights into the RVOT’s role in arrhythmogenesis and procedural success. Additionally, advancements in imaging technology, such as real-time MRI or intravascular ultrasound, may offer new opportunities to visualize and characterize RVOT anatomy dynamically during interventions.

## Conclusions

This study provides a detailed definition, along with anatomical and structural analysis of the RVOT, highlighting its complex morphology and its significant role in cardiac functionality and clinical interventions. The RVOT has a truncated cone shape with the elliptical RVOT base being almost two times larger than its distal boundary (the basal ring of pulmonary root). The RVOT may be divided into free-standing thin wall subpulmonary infundibulum and septal component. The RVOT wall thickness demonstrated significant differences both among the respective RVOT walls and across the RVOT levels, with the posterior wall being the thickest and the anterior and left walls demonstrating consistent thinner profiles. Endocardial roughness of the RVOT may be observed in all hearts within the subpulmonary infundibulum, but are no evenly distributed. By delivering and analyzing key geometric parameters of the RVOT components our research may enhance the understanding of the structural challenges and potential complications associated with cardiac procedures, including catheter ablations, lead implantations, and structural interventions within the pulmonary valve and trunk.

## Electronic supplementary material

Below is the link to the electronic supplementary material.

Supplementary Material 1

## Data Availability

All data generated or analysed during this study are included in this article. Further enquiries can be directed to the corresponding author.
