# Supplementary material for: Right ventricular outflow tract morphology and its clinical significance for invasive procedures
Source: Sci Rep. 2025 Jul 9;15:24694. doi: 10.1038/s41598-025-06663-w (PMC12241634; doi:10.1038/s41598-025-06663-w)
Supplement: Supplementary file 1 — Supplementary Material 1 [file 41598_2025_6663_MOESM1_ESM.docx]

**Supplementary Table 1 – Whole-wall, myocardial, and epicardial adipose tissue thickness of the right ventricular outflow tract (RVOT) walls presented as mean ± SD [range: min–max], with statistical comparisons between different measurement points (ANOVA).**

| Parameter | Level | **RVOT wall** | | | | **p-value for comparison between walls** | | | | | | | **p-value for comparison between levels** | | | |
| --- | --- | --- | --- | --- | --- | --- | --- | --- | --- | --- | --- | --- | --- | --- | --- | --- |
|  |  |  |  |  |  | ANOVA | 1 vs. 2 | 1 vs. 3 | 1 vs. 4 | 2 vs. 3 | 2 vs. 4 | 3 vs. 4 | ANOVA | A vs. B | A vs. C | B vs. C |
|  |  | anterior  (1) | posterior (2) | left  (3) | right  (4) |  |  |  |  |  |  |  |  |  |  |  |
| Whole-wall  thickness (mm) | Proximal (A) | 5.7 ± 2.3  [1.4-23.0] | -* | 5.6 ± 2.4  [2.3-18.3] | 7.9 ± 2.6  [2.3-15.5] | <0.001 | - | 1.000 | <0.001 | - | - | <0.001 | <0.001 | <0.001 | 1.000 | 0.003 |
|  | Middle  (B) | 6.2 ± 2.0  [1.6-15.5] | 10.3 ± 3.8  [1.6-20.0] | 6.0 ± 2.2  [2.4-14.8] | 7.4 ± 2.7  [1.9-17.2] | <0.001 | <0.001 | 1.000 | <0.001 | <0.001 | <0.001 | <0.001 |  |  |  |  |
|  | Distal  (C) | 5.9 ± 1.8  [2.0-12.5] | 8.7 ± 3.5  [1.6-17.5] | 6.1 ± 2.2  [2.3-18.2] | 8.6 ± 2.7  [3.5-17.3] | <0.001 | <0.001 | 1.000 | <0.001 | <0.001 | <0.001 | <0.001 |  |  |  |  |
| Myocardial  thickness (mm) | Proximal(A) | 4.7 ± 1.4  [1.4–12.1] | -* | 4.0 ± 1.4  [1.4-9.8] | 5.4 ± 1.6  [1.9-10.4] | <0.001 | - | 0.002 | 0.013 | - | - | <0.001 | <0.001 | <0.001 | <0.001 | 0.003 |
|  | Middle (B) | 4.7 ± 1.3  [1.6-14.3] | 10.3 ± 3.8  [1.6-20.0] | 4.3 ± 1.2  [1.5-7.4] | 5.3 ± 1.5  [2.0-10.7] | <0.001 | <0.001 | 0.704 | 0.161 | <0.001 | <0.001 | <0.001 |  |  |  |  |
|  | Distal (C) | 4.2 ± 1.2  [1.8-8.8] | 8.7 ± 3.5  [1.6-17.5] | 4.3 ± 1.2  [1.8-8.7] | 4.2 ± 1.4  [1.7-8.5] | <0.001 | <0.001 | 1.000 | 1.000 | <0.001 | <0.001 | 1.000 |  |  |  |  |
| Epicardial adipose tissue thickness (mm) | Proximal (A) | 1.0 ± 1.6 [0.0-10.9] | none | 1.7 ± 1.7  [0.0-11.0] | 2.6 ± 2.0  [0.0-11.3] | <0.001 | - | 0.004 | <0.001 | - | - | <0.001 | <0.001 | <0.001 | <0.001 | 0.451 |
|  | Middle  (B) | 1.6 ± 1.5  [0.0-7.9] | none | 1.8 ± 1.7  [0.0-12.0] | 3.2 ± 2.3  [0.0-11.9] | <0.001 | - | 1.000 | <0.001 | - | - | <0.001 |  |  |  |  |
|  | Distal  (C) | 1.7 ± 1.3  [0.0-6.3] | none | 1.9 ± 1.8  [0.0-14.3] | 3.4 ± 2.1  [0.0-12.8] | <0.001 | - | 1.000 | <0.001 | - | - | <0.001 |  |  |  |  |

* - posterior wall of the RVOT corresponds to the interventricular septum and RVOT septal component, where not all measurements were possible at all levels and where epicardial adipose tissue is absent.
